# Thumb Reconstruction Using Osseointegrated Dental Implants for Prosthetic Retention Following Gunshot Amputation: A Case Report

**DOI:** 10.7759/cureus.107403

**Published:** 2026-04-20

**Authors:** Ahmad Alzoubi, Ahmad Almigdad, Iasmina Alhiasat

**Affiliations:** 1 Department of Orthopaedics, Royal Medical Services, Amman, JOR; 2 Department of Orthopedics, Royal Medical Services, Amman, JOR; 3 Department of Dentistry, Royal Medical Services, Amman, JOR

**Keywords:** amputation, dental implant, osseointegration, prosthetic retention, thumb

## Abstract

Thumb amputations severely compromise hand function, particularly grip and opposition. Osseointegration, originally developed for dental implantation, has become a promising method for directly anchoring prostheses to bone. This technique uses a two-stage protocol: first, a dental implant is placed into the medullary canal and allowed to integrate with the surrounding bone; once stable osseointegration is confirmed, a skin-penetrating abutment and custom prosthesis are attached. This approach provides secure fixation even when bone stock is limited and offers several advantages over conventional prosthetics, including improved retention, stronger grip and pinch function, increased range of motion, and enhanced osseoperceptive feedback.

We present the case of a 38-year-old male who sustained a gunshot injury resulting in amputation at the base of the proximal phalanx of the right thumb. The first stage of reconstruction included metacarpophalangeal joint arthrodesis and first web-space widening using a four-flap Z-plasty. A dental implant was placed across the fusion site to provide both fixation for the arthrodesis and a stable anchoring platform for a custom prosthesis. This case suggests that osseointegrated dental implants may provide a feasible retention option for thumb prosthetic reconstruction, particularly when conventional reconstructive strategies are limited or contraindicated.

## Introduction

Digital amputations, particularly those involving the thumb, result in substantial functional and aesthetic impairment due to the thumb’s essential role in pinch, grip, and overall hand dexterity [1]. Reconstruction following traumatic thumb amputation remains surgically challenging, especially in delayed presentations or in the presence of inadequate soft tissue coverage and a shortened residual stump [2]. Although established reconstructive options-such as replantation, toe-to-thumb transfer, and pollicization-can achieve satisfactory outcomes, they are not always feasible due to patient-related factors, resource limitations, or local tissue conditions. In this context, osseointegration, originally developed for dental applications, has emerged as a promising technique for directly anchoring prostheses to bone [3]. In this approach, a two-stage implant protocol is employed: first, the implant is inserted into the medullary canal and allowed to osseointegrate; then, following confirmation of stable bone integration, a skin-penetrating abutment and custom prosthesis are attached. This method provides stable fixation even in cases with limited bone stock and has been reported in the literature to offer potential advantages over conventional prosthetics, including improved retention, functional grip and pinch strength, increased range of motion, and osseoperceptive feedback [4,5]. However, this technique is not without limitations, as potential complications include infection at the skin-implant interface, implant loosening or failure, soft tissue irritation, and the need for meticulous long-term care, while evidence in upper limb applications, particularly in digital reconstruction, remains limited and is largely derived from small case series. Nevertheless, for patients who are not candidates for complex reconstructive procedures, osseointegrated implants may offer a valuable alternative, with the potential to restore both function and psychosocial well-being. This report describes a case of delayed thumb reconstruction using a hydroxyapatite-coated dental implant combined with metacarpophalangeal arthrodesis and web-space widening, with satisfactory functional and aesthetic outcomes.

## Case presentation

**Figure 1:**
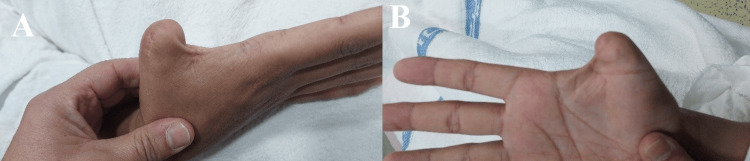
Clinical photographs of the patient before the treatment (A, B) Clinical photographs demonstrating thumb amputation at the level of the proximal phalanx, with a narrowed first web space.

**Figure 2:**
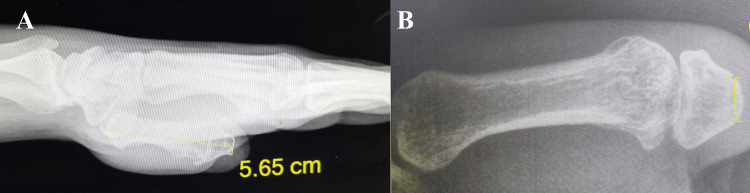
Radiographic images of the patient before the treatment (A, B) Radiographic images showing amputation at the base of the proximal phalanx of the thumb.

A 38-year-old male patient sustained a traumatic amputation of the right thumb at the level of the base of the proximal phalanx as a result of a gunshot injury, which occurred two years prior to presentation. Clinical examination revealed a shortened thumb stump with a narrowed first web space and no detectable movement at the metacarpophalangeal joint (MCPJ) (Figure 1). Radiographic evaluation demonstrated a short remnant of the proximal phalanx base (Figure 2).

The treatment options considered included either excision of the residual proximal phalanx with dental implant insertion into the first metacarpal or arthrodesis between the remaining phalanx and metacarpal with simultaneous implant placement. The latter approach, although associated with a longer recovery period and potential risk of fusion failure, offered the advantage of improved bone purchase due to the narrow intramedullary canal of the phalanx compared to the metacarpal head, potentially resulting in more secure osseointegration.

**Figure 3:**
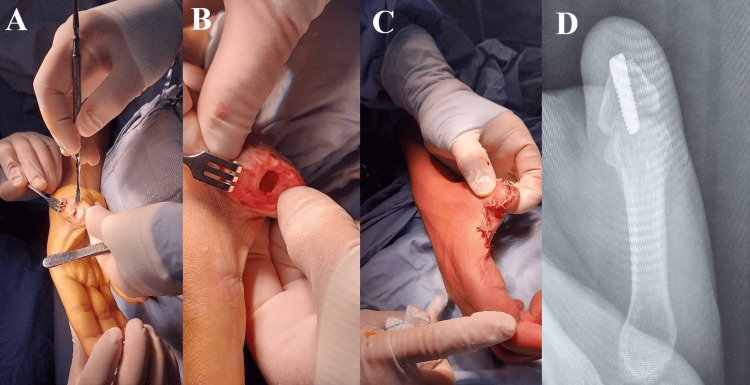
Intraoperative images (A, B) Intraoperative images demonstrating the first stage, including preparation of the bone for insertion of a dental implant. (C) Widening of the first web space to improve prosthesis fitting and thumb grip. (D) Radiographic image showing the dental implant in position.

After a thorough discussion of the risks and benefits, the patient consented to proceed with arthrodesis between the residual proximal phalanx and the metacarpal, combined with simultaneous web space widening during the first surgical stage, in order to preserve thumb length and improve functional grip. This step was also intended to optimize the residual stump and enhance future prosthetic stability, even in the event of prosthesis loosening or removal (Figure 3).

Surgery was performed under regional anesthesia with tourniquet control. Web space widening was achieved through a four-flap Z-plasty. A transverse skin incision was made at the distal end of the thumb stump, followed by layer-by-layer dissection and identification of the residual bone. Any bony prominences and remaining articular cartilage at the metacarpophalangeal joint were removed. An allograft was applied at the arthrodesis site, which was temporarily stabilized using Kirschner wires.

The intramedullary canal of the remnant phalanx and metacarpal was then prepared using a pilot drill and confirmed under fluoroscopy. Sequential drills from the dental implant kit were used to gradually widen and deepen the canal until satisfactory bone engagement was achieved. A hydroxyapatite-coated titanium dental implant was inserted and covered with a cover screw. The implant also served as a compression screw to provide definitive fixation across the fusion site. The surgical wound was then closed in layers. Postoperatively, the patient was monitored for wound healing and progression of bone fusion. Complete fusion of the arthrodesis site was confirmed by X-ray at three months.

**Figure 4:**
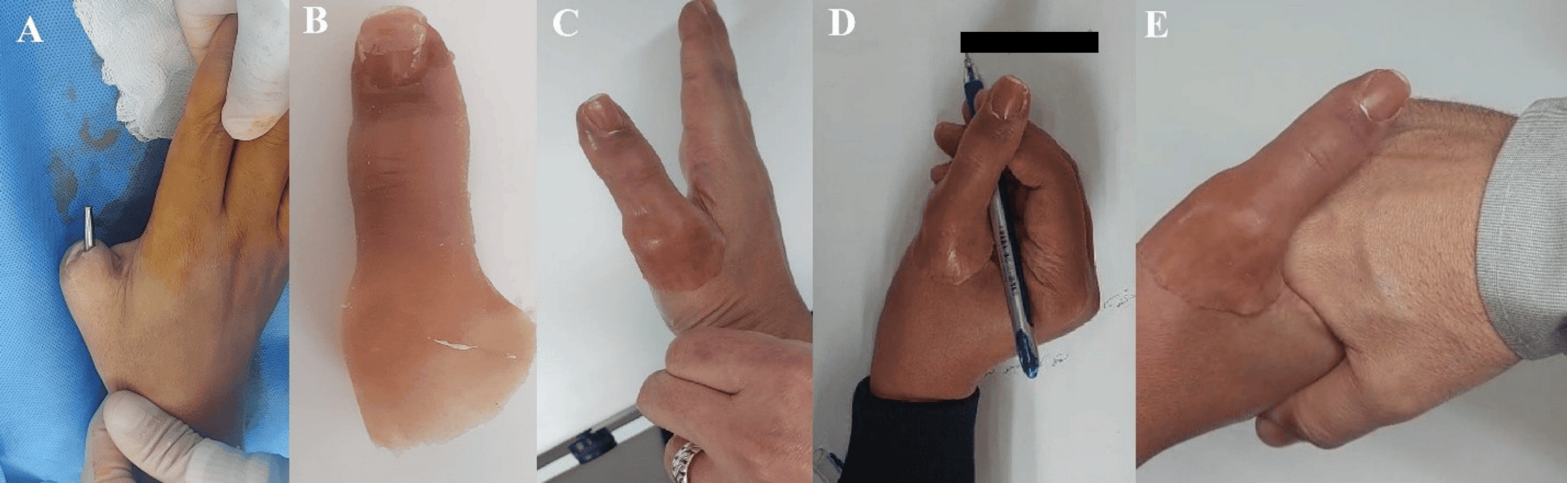
Second stage of the procedure (A) Second stage of the procedure demonstrating abutment fixation to the dental implant. (B) Fabrication of the silicone prosthesis. (C) Retained silicone prosthesis in position. (D, E) Retained prosthesis demonstrating functional and cosmetic improvement.

In the second stage, the skin over the implant was reopened, and healing abutments were attached. Following a two-week healing phase, impression copings were placed on the implants, and final impressions were obtained using silicone impression material to fabricate the custom prosthesis (Figure 4). The patient expressed high satisfaction with the cosmetic and functional outcomes, demonstrating improved grip and pinch strength, along with enhanced proprioceptive and tactile sensibility.

## Discussion

Finger amputations, particularly of the thumb or index finger, profoundly impact both hand function and psychological well-being, given these digits’ essential roles in grasping and fine motor tasks [6]. Surgical approaches such as toe-to-hand transfers, pollicization, and local flaps can restore function; however, these procedures are technically demanding and may yield suboptimal aesthetic outcomes [7,8]. Even when functional gains are achieved, discrepancies in shape, size, and color may reduce patient satisfaction. In comparison, prosthetic rehabilitation-although lacking biological integration and true sensory feedback-emerges as a critical alternative for individuals in whom reconstruction is not possible, is declined, or has failed, offering acceptable restoration of appearance and basic functional support.

Conventional silicone prostheses offer some cosmetic benefits, but their functional limitations are considerable. They often lack secure fixation, provide no sensory feedback, and are associated with problems such as perspiration buildup, poor skin contact, and frequent need for replacement [9]. In contrast, osseointegrated implants offer a promising alternative by anchoring the prosthesis directly to the bone. This approach improves prosthetic retention and control and may provide a degree of tactile feedback, known as osseoperception [5].

Our case is consistent with existing literature, suggesting that osseointegrated dental implants can effectively retain finger and thumb prostheses, offering stable, functional, and aesthetically acceptable outcomes. Similar findings have been reported by Aydin et al. [10] and Sierakowski et al. [11], with high levels of patient satisfaction, stable prosthetic retention, and reliable performance in daily tasks. Li et al. [12] compared osseointegration with digital replantation, reporting that while osseointegration showed slightly reduced sensory and motion scores, it provided comparable overall satisfaction and function. These findings support its role as a valuable alternative, particularly in delayed presentations or when complex microsurgical reconstruction is not feasible.

This technique may be adaptable in selected cases and can be combined with complementary procedures such as arthrodesis or web space widening to optimize functional outcomes. Implant positioning should be individualized based on residual bone stock, allowing anchorage at either the phalangeal or metacarpal level when appropriate, which may extend its applicability across different amputation patterns. Osseointegration can be performed using either a one-stage or a two-stage surgical technique. Both approaches have demonstrated favorable clinical outcomes. The one-stage procedure offers the advantage of reduced operative time and accelerated rehabilitation. However, the two-stage technique is preferable when initial implant stability is inadequate or when soft-tissue coverage is compromised, as was the case in the present study [13].

The primary drawback of osseointegration is that it often requires staged surgical procedures, which may increase the overall risk of infection and prolong the rehabilitation period. Reported complication rates vary across studies but commonly include skin-implant interface infection, abutment loosening, soft-tissue irritation, and cold intolerance; less frequently, failure of osseointegration has also been described. However, most complications are manageable and rarely compromise long-term prosthetic function [14]. Successful outcomes depend on careful patient selection, adequate bone quality, and the establishment of realistic patient expectations.

## Conclusions

Osseointegrated dental implants in this case provided a promising solution for reconstruction of thumb amputation when replantation or complex microsurgical reconstruction was not feasible, with the bone-anchored prosthesis offering improved retention, functional control, and a degree of sensory feedback via osseoperception, thereby addressing key limitations of conventional socket-based prostheses; the patient reported improved grip, pinch strength, daily function, and satisfactory cosmetic outcome. However, as reported in the literature, potential complications such as skin-implant interface infection, soft tissue irritation, implant loosening, and, less frequently, failure of osseointegration should be considered, although none were encountered in this case.

Although staged surgery introduces inherent risks, these are generally manageable with appropriate technique and follow-up. The adaptability of the method to different amputation levels and its compatibility with adjunctive procedures may broaden its potential application. This case suggests that, with careful patient selection and planning, osseointegration may represent a viable option for functional and aesthetic rehabilitation after traumatic digital loss.
